# Prostate cancer treated with brachytherapy; an exploratory study of dose-dependent biomarkers and quality of life

**DOI:** 10.1186/s13014-017-0792-1

**Published:** 2017-03-14

**Authors:** Sarah O. S. Osman, Simon Horn, Darren Brady, Stephen J. McMahon, Ahamed B. Mohamed Yoosuf, Darren Mitchell, Karen Crowther, Ciara A. Lyons, Alan R. Hounsell, Kevin M. Prise, Conor K. McGarry, Suneil Jain, Joe M. O’Sullivan

**Affiliations:** 1 Centre of Cancer Research and Cell Biology, Queen’s University Belfast, BT7 1NN Belfast, UK; 2 Radiotherapy Physics, Northern Ireland Cancer Centre, Belfast Health and Social Care Trust, Belfast, UK; 3 Clinical Oncology, Northern Ireland Cancer Centre, Belfast Health and Social Care Trust, Belfast, UK

**Keywords:** Permanent prostate brachytherapy, DNA damage biomarkers (γH2AX and 53BP1), EPIC, Sector analysis

## Abstract

**Background:**

Low-dose-rate permanent prostate brachytherapy (PPB) is an attractive treatment option for patients with localised prostate cancer with excellent outcomes. As standard CT-based post-implant dosimetry often correlates poorly with late treatment-related toxicity, this exploratory (proof of concept) study was conducted to investigate correlations between radiation − induced DNA damage biomarker levels, and acute and late bowel, urinary, and sexual toxicity.

**Methods:**

Twelve patients treated with ^125^I PPB monotherapy (145Gy) for prostate cancer were included in this prospective study. Post-implant CT based dosimetry assessed the minimum dose encompassing 90% (D_90%_) of the whole prostate volume (global), sub-regions of the prostate (12 sectors) and the near maximum doses (D_0.1cc_, D_2cc_) for the rectum and bladder. Six blood samples were collected from each patient; pre-treatment, 1 h (h), 4 h, 24 h post-implant, at 4 weeks (w) and at 3 months (m). DNA double strand breaks were investigated by staining the blood samples with immunofluorescence antibodies to γH2AX and 53BP1 proteins (γH2AX/53BP1). Patient self-scored quality of life from the Expanded Prostate Cancer Index Composite (EPIC) were obtained at baseline, 1 m, 3 m, 6 m, 9 m, 1 year (y), 2y and 3y post-treatment. Spearman’s correlation coefficients were used to evaluate correlations between temporal changes in γH2AX/53BP1, dose and toxicity.

**Results:**

The minimum follow up was 2 years. Population mean prostate D_90%_ was 144.6 ± 12.1 Gy and rectal near maximum dose D_0.1cc_ = 153.0 ± 30.8 Gy and D_2cc_ = 62.7 ± 12.1 Gy and for the bladder D_0.1cc_ = 123.1 ± 27.0 Gy and D_2cc_ = 70.9 ± 11.9 Gy. Changes in EPIC scores from baseline showed high positive correlation between acute toxicity and late toxicity for both urinary and bowel symptoms. Increased production of γH2AX/53BP1 at 24 h relative to baseline positively correlated with late bowel symptoms. Overall, no correlations were observed between dose metrics (prostate global or sector doses) and γH2AX/53BP1 foci counts.

**Conclusions:**

Our results show that a prompt increase in γH2AX/53BP1foci at 24 h post-implant relative to baseline may be a useful measure to assess elevated risk of late RT − related toxicities for PPB patients. A subsequent investigation recruiting a larger cohort of patients is warranted to verify our findings.

**Electronic supplementary material:**

The online version of this article (doi:10.1186/s13014-017-0792-1) contains supplementary material, which is available to authorized users.

## Background

Prostate cancer is the most commonly diagnosed male cancer in the UK with an incidence of 103 cases per 100,000 (European age-standardised rates, 2010) and almost 47,300 patients were diagnosed in the UK alone in 2013 (Cancer Research UK). Initial treatment options include active surveillance [1], androgen deprivation therapy (ADT) [2], radical prostatectomy [3], external beam radiotherapy (EBRT) and brachytherapy with a patient’s Gleason score and tumour staging primarily dictating which treatment modality is optimal. For localised prostate cancer (T1-T2, Gleason score ≤ 7 and PSA < 20 ng/ml), low-dose-rate permanent prostate brachytherapy (PPB) is an attractive choice of treatment due to its convenience, favourable dose distribution to normal tissues, high success rate with regards to both progression free and overall survival [4, 5] and its potential higher likelihood of potency preservation when compared to prostatectomy [6].

**Table 1:** Patient basic baseline characteristics

Patient #	Age at diagnosis	PSA (ng/mL)	Gleason (primary + secondary)	Risk group
1	60	9.2	3 + 4	2
2	58	9.7	3 + 4	2
3	60	10.9	3 + 3	2
4	55	8.6	4 + 3	2
5	55	9.2	3 + 3	1
6	51	8.7	3 + 4	2
7	54	6.7	4 + 3	2
8	74	7.4	4 + 3	2
9	57	7.7	3 + 4	2
10	65	14.5	4 + 3	2
11	53	3.9	3 + 4	2
12	57	11.6	3 + 4	2

Risk group 1 = low risk, 2 = intermediate risk

Current post-implant dosimetric evaluation for PPB is calculated using CT derived models while patient outcomes and quality of life (QoL), in the form of function and bother, can be measured using patient − reported outcome measures such as the Expanded Prostate Cancer Index Composite (EPIC) questionnaire [7]. The use of blood − derived radiation biomarkers have provided in-vivo detection of irradiation for large volume acute radiation exposures [8–10], however their utility for small volume prolonged PPB exposures within brachytherapy has yet to be established. The histone H2AX is phosphorylated to γH2AX at the sites of radiation induced DNA double − strand breaks (DSBs) [11], where it co-localises with the DNA repair protein 53BP1. Loss of γH2AX/53 PB1 over time correlates well with the kinetics of DSBs repair, and has been extensively used to probe the kinetics of repair in a range of conditions [11–15]. γH2AX/53BP1 form distinct nuclear foci which can be readily detected and quantified using immunofluorescence microscopy. For acute doses, a minimum sensitivity down to 5 mGy was detected in patients undergoing a chest CT by comparing pre- and post- CT γH2AX foci numbers in peripheral blood lymphocytes [16]. With low background numbers of DSBs and a well characterised radiation response, peripheral blood lymphocytes are well established as an ideal ex-vivo cell model to use for biological dosimetry.

The level of detectable DNA damage in the lymphocyte population, which contains cells constantly circulating throughout the peripheral blood and also stationary in reservoirs throughout the body such as lymph nodes and spleen, may not correlate simply to the equivalent whole body dose delivered to the patient. There is also increasing evidence that doses received by the prostate and surrounding organs such as the bladder and rectum will display varying degrees of inter- and intra-patient variability [17, 18]. Whether radiation induced DNA damage to the lymphocyte population is dependent on where doses are targeted rather than simply the total dose delivered to all tissues is currently poorly understood.

The primary objective of this study was to investigate and quantify, for the first time, the induction and persistence of γH2AX/53BP1 foci in peripheral blood lymphocytes in patients undergoing low-dose-rate PPB for prostate cancer. Furthermore, we tested the hypothesis that levels of biological DNA damage biomarkers relative to baseline would correlate with patient reported QoL outcome measures determined using the EPIC questionnaire.

## Methods

### Patient cohort

Following ethical approval, 12 patients eligible for treatment with PPB for prostate cancer were identified and recruited for this study. Patients’ baseline characteristics are presented in Table 1. Written information was provided and potential subjects were given at least 7 days to decide upon participation. Inclusion criteria were as follows; patients with histologically confirmed adenocarcinoma of the prostate, who elected for low-dose-rate permanent prostate brachytherapy, WHO performance status 0 – 2, life expectancy of at least 12 months, age ≥ 18 years and a willingness to co-operate with follow-up. Exclusion criteria were; chronic bowel disorder or previous bowel surgery, rheumatoid arthritis, auto immune disease, an active bacterial, viral or fungal infection, renal impairment, diabetes or previous radiotherapy.

### Planning

**Fig. 1:**
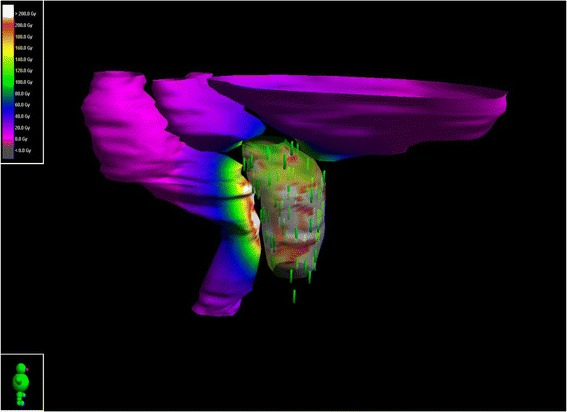
Typical dose distribution following ^125^I prostate brachytherapy. The rectum, prostate and bladder have been contoured on a CT scan 4 weeks after brachytherapy and reconstructed in 3D. Brachytherapy seeds within the prostate are highlighted green and a colourwash representing total dose has been applied to the surface of each organ

Prior to implantation, all patients underwent a detailed prostate volume study using a bi-planar trans-rectal probe 8848 (BK Medical Systems Inc. Washington, MA). All implants were planned using ^125^I stranded radioactive seeds with a reference air kerma rate of 0.458 – 0.496 U (U –μGyh^−1^m^2^) [19, 20]. The half-life of Iodine 125 is 59.43 days. The American Association of Physicists in Medicine Task Group 43 (TG-43 update) formalism was used in the planning and calculation of the final dosimetry [21]. All treatment plans were created to meet the objectives and constraints recommended by ESTRO/EAU/EORTC guidelines [22]. A Variseed 8.0 (Varian Medical Systems, Charlottesville, VA) treatment planning system (TPS) was used for both pre- and post- implant planning. The minimum prescription dose was 145 Gy for all patients, representing the planned dose that will encompass the entire target volume. The dose inside the target volume will be >145Gy, therefore planning constraints are used; no more that 55% of the target volume may receive ≥150% of dose (217.5Gy) and the volume receiving ≥200% (290Gy) should be limited to ≤ 20%. Post-implant CT scans (2.5 mm slice thickness) were obtained four weeks after implantation. CT scans were then transferred to the TPS and the structures including prostate, bladder and rectum were delineated. Although a pre-implantation plan is created to ensure 100% dose to the planning target volume, the practicalities of inserting needles into the prostate together with post-surgical oedema and seed migration can result in differences between the intended and delivered dose [23–25]. With this information, a dose distribution for each organ can be created for each patient; Fig. 1 is a typical example.

Post-implant dosimetry was evaluated for whole prostate (global) minimum dose to 90% of prostate (D_90%_) and the maximum dose, 0.1 cc (D_0.1cc_(Gy)) and 2 cc (D_2cc_ (%)), received by the rectum and bladder. These point doses represent the highest dose delivered to the identified organ at risk and are described as by either absolute dose in Gy or as percentage of the prescribed dose and allow uniform comparison of acute and late toxicity to organ at risk dosimetry. In addition, sector analysis of 12 prostatic regions (sectors) was conducted. As described in more details in previous publications [25, 26], sector analysis involved dividing the prostate into three equal lengths along the cranio-caudal axis to create base, mid-gland and apex, then sub-dividing with a vertical plane and a horizontal plane creating right/left anterior and right/ left posterior sectors (also see Additional file 1: Figure S1). D_90%_ was then determined for each sector of the prostate.

### γH2AX and 53BP1 detection

In all the cases, peripheral venous blood was sampled in an EDTA tube and placed immediately on ice prior to transfer for analysis. For each patient, a sample was taken prior to brachytherapy to acquire a baseline and succeeding samples were gathered at 1 h, 4 h, 24 h, 4 weeks and 3 months post-seed implantation (a total of 6 blood samples for each patient). Blood samples at 4 weeks were always collected just before the acquisition of post-implant CT scans.

### Separation of lymphocytes for γH2AX /53BP1 foci analysis

4 ml of whole blood was pipetted onto 3 ml of Ficoll-Plaque (GE Healthcare) and centrifuged at 4 oC, 400G, for 30 min. 1 ml of separated lymphocytes were aspirated and mixed with 10 ml of sterile phosphate buffered saline (PBS) to wash. The lymphocyte suspension was centrifuged at 4 oC, 100 g, for 20 min to form a cell pellet. The remaining PBS was decanted and the pellet re-suspended with 1 ml of fresh sterile PBS.

### Fixation, staining of PBLs for γH2AX and 53BP1 foci

100 μl of re-suspended lymphocytes were loaded onto Superfrost Plus (VWR international) glass slides by cyto-spinning at 500 g for 10 min. The cells were then fixed with 4% formaldehyde at room temperature for 10 min. Each slide was then washed 3 times with PBS. The cells were permeabilized with a 0.5% (v/v) solution of Triton-X/PBS for 10 min. Blocking was achieved with 1% BSA in PBS for 1 hour at room temperature. Cells were then incubated for 1 h at room temperature with a solution of 1:5000 Mouse anti SER 139 H2AX (Millipore) and rabbit anti 53BP1 (Novus biologicals) in 0.05% Triton-X/PBS. Cells were then washed with 0.05% Triton-X/PBS. Secondary antibodies 1:2000 of goat anti-mouse Alexa-fluro 488 (Invitrogen) and goat anti rabbit 53BP1 in 0.05% Triton-X/PBS were applied for a further 1 h at room temperature. Cells were then washed with 0.05% Triton-X/PBS and air dried at room temperature. The slides were mounted with Prolong Antifade Gold with DAPI (Life Technologies) and coverslip.

### Visualisation and counting of foci

Cells were visualised (by a single observer) using a Nikon fluorescent microscope at x63 magnification. Lymphocytes were identified by morphology after DAPI staining, other cell types were excluded from counting. γH2AX foci co-localising with 53BP1 foci were counted by eye in 200 cells for each time point.

### Health related quality of life endpoints

Heath related QoL was assessed using the Expanded Prostate Cancer Index Composite (EPIC) instrument [7]. The EPIC questionnaire is divided into domains related to bowel, urinary, sexual and hormonal toxicities. Each domain is divided into functional and bother scores which can be combined to produce a summary score. For this study, bowel, urinary and sexual summary scores were assessed. A score of 100 indicates no problem; lower values indicate complications. For each patient, EPIC scores were obtained at baseline (pre brachytherapy), 1 month (m) post-brachytherapy, 3 m, 6 m, 9 m, 1 year (y), 2y and 3y. Primary endpoints were bowel-, urinary- and sexual- related toxicities after brachytherapy. Change-from baseline analysis was conducted using the EPIC scores. Acute toxicity was defined as those events (i.e. decrease of score from baseline) that presented within the first 3 months following treatment. Late toxicity was defined as events that developed at any time after treatment and not resolved at 2–3 years after treatment.

### Statistical analysis

All correlation analysis was conducted in R (version 3.2.0) using Spearman’s correlation testing at a significance level p ≤0.05. Although multiple comparisons were conducted, no corrections were applied due to the exploratory/feasibility nature of this study (also see related section in the discussion). Moreover, to test if foci distributions corresponded to a Poisson distribution at each time-point, *χ*2 testing was used with a significance threshold of *p =* 0.05.

## Results

### γH2AX and 53BP1 foci induction

**Fig. 2:**
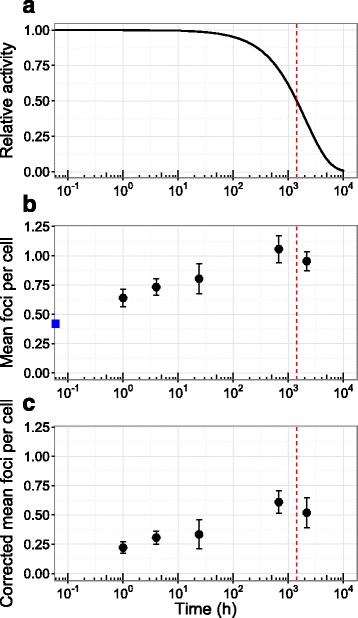
**a**) Relative activity of ^125^I as a function of time post-seed-implantation. **b**) mean foci number per cell in patients' peripheralblood lymphocytes (the blue square on the vertical axis indicates mean background counts) and corrected for background (**c**). The error bars represent the standard error and the red dashed line represents the ^125^I half-life of 1426.3 h (59.43 days)

Prior to implantation of ^125^I seeds into the prostate, mean (±SD) background foci (co-localising γH2AX/53BP1) was measured at 0.42 ± 0.20 foci per cell, ranging from 0.11 to 0.72 (Fig. 2b). A Shapiro-Wilk test confirmed the data was normally distributed (w = 0.943, *p = 0.540*). Following seed implantation, γH2AX/53BP1 foci numbers were significantly elevated as early as 1 h post-implantation (paired *t*-test *p < 0.001*), and remained significantly higher than background levels at 4 and 24 h, 4 weeks and 3 months post-implantation (Fig. 2b). Fig. 2c shows the mean number of foci per cell corrected for background.

The highest foci numbers were observed 4 weeks (672 h) after irradiation, at 1.1 ± 0.1 foci per cell. This value is more than 2.5-fold above background foci levels (0.4 ± 0.1) and is significantly higher than 24 h values (paired *t*-test *p = 0.02*), suggesting that brachytherapy acts as a significant additional source of genetic stress in these lymphocytes. Based on ex vivo lymphocyte studies [11] showing an average of 15.5 foci induced per Gy with an average half-life of 2 h, an average of 0.7 excess foci per cell corresponds to an average dose-rate to the lymphocyte population of 4.5 cGy/h. Interestingly, this dose rate is comparable to the initial dose rate delivered to the prostate, 7.1 cGy/h (Additional file 2: Figure S2), despite the majority of lymphocytes not being within the exposed volume at any given time. This suggests either the impact of other stresses on lymphocyte DNA damage levels, or the formation of DSBs with a long half-life. This is further supported by the observation that γH2AX/53BP1 foci numbers continued to rise at 4 weeks (672 h) even after reduction in seeds activity due to natural decay (Fig. 2a), and showing only limited (statistically insignificant) decline at 3 months despite source activity falling by almost three-quarters.

**Fig. 3:**
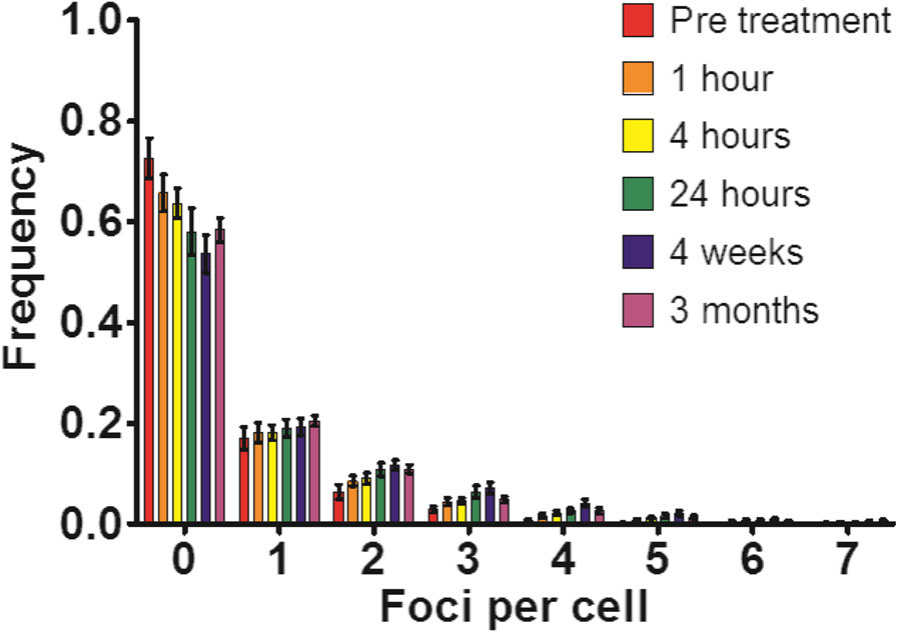
Foci distribution counts in patients’ peripheral blood lymphocytes pre- and post- seed implantation. The error bars represent the standard error

As the increase in mean foci counts over time may have been due to an increased proportion of observed cells with foci or greater foci numbers in irradiated cells, the relative frequency distribution of foci per lymphocyte over time was plotted (Fig. 3). It can be seen that, at all time-points post-implantation, there is a gradual increase in both the number of hit cells, and the number of cells at each foci level, indicating some increase in damage in large number of cells rather than the presence of a small subpopulation of highly damaged cells. At no time point did more than 1% of all cells show more than 7 foci. However, while this damage may have been expected to follow a Poisson distribution, all foci distributions are significantly different to that predicted by a Poisson distribution (*p* < 0.0001), with an excess of undamaged cells, suggesting some degree of mixing between populations exposed to different stresses.

### QoL scores over time

**Fig. 4:**
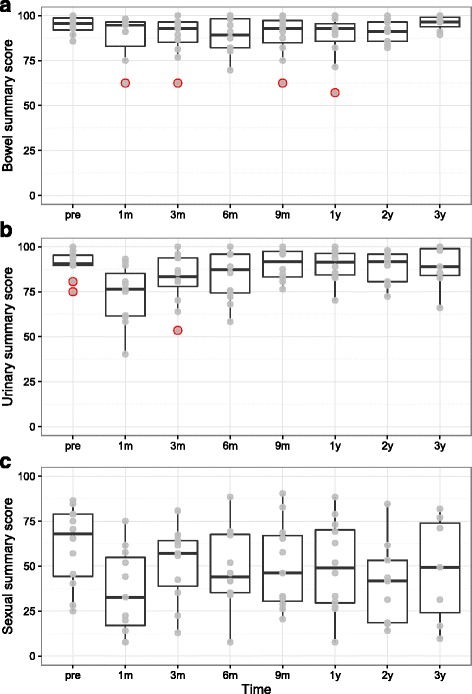
Changes in quality-of-life scores over time for each domain studied on the Expanded Prostate Cancer Index Composite (EPIC) questionnaire. EPIC scores range from 0 to 100, with higher values representing a more favorable health-related quality of life. In the figure median with inter-quartile range IQR (boxes), and ± 1.5*IQR (whiskers) are presented. Dots are different data points and red dots are outliers

As demonstrated in Fig. 1, the main neighbouring organs at risk which receive high dose in prostate brachytherapy are the prostate, urethra, rectum and bladder and toxicities observed in patients undergoing brachytherapy are usually related to these organs. QoL in terms of patients’ bowel, urinary and sexual health was measured using the EPIC questionnaire before seed-implantation and up to three years post-treatment. Increased toxicities were observed post-implant peaking at 6 m for bowel symptoms (Fig. 4a), and at 1 month post-implant for both urinary (Fig. 4b) and sexual (Fig. 4c) symptoms.

### Global analysis

Population median prostate global D_90%_ was (median (range)) 144.4 Gy (range: 125.4 Gy −160.3 Gy). The median of the highest point dose within the contoured rectum represented by D_0.1cc_ was 145.2 Gy (range: 112.5 Gy – 212.5 Gy) and the highest dose delivered to a 2 cc of the rectum (D_2cc_) was 62.0% (range: 42.4% –84.8%). Bladder median D_0.1cc_ was 123.0 Gy (81.3 Gy − 180.3 Gy) and D_2cc_ =70.4% (54.0% − 91.7%)_._

### Foci count, EPIC, sector analysis and dosimetric parameters: initial exploratory correlation analysis

**Fig. 5:**
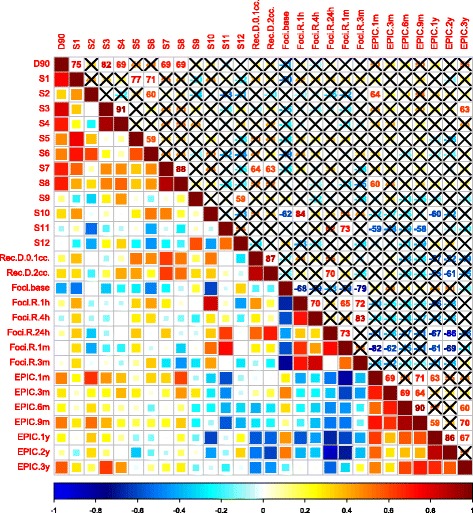
Bowel-related cross-correlation matrix of Spearman’s correlation coefficients (ρ). Upper triangle; ρ values with insignificant correlation (*p* ≤ 0.05) marked with an X. Lower triangle; colour coded coefficients. S(x) = prostatic sector number ‘x’, Rec = rectum, D_0.1cc_ and D_2cc_ = the doses received by the hottest 0.1 cc and 2 cc of the volume, respectively. Foci base = γH2AX/53BP1 foci count at baseline (pre-implant (t = 0 h)). Foci R t = ratio of foci count at time point t relative to baseline (0 h). EPIC(x) = change in EPIC score from baseline; positive values reflecting improvement, negative values deterioration and 0 no change. * For space consideration all ρ values are presented as percentages

**Fig. 6:**
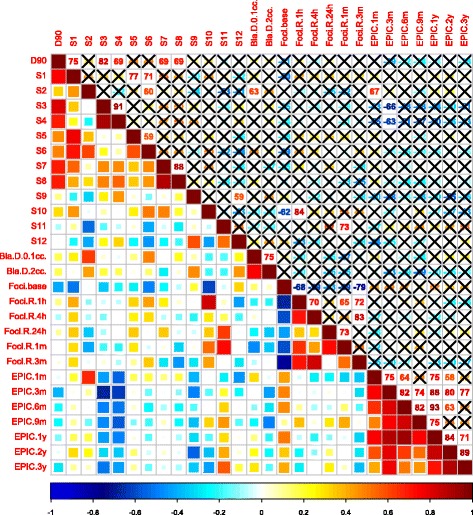
Bladder-related cross-correlation matrix of Spearman’s correlation coefficients. (ρ). Upper triangle; *ρ* values with insignificant correlation (*p* ≤ 0.05) marked with an X. Lower triangle; colour coded coefficients. S(x) = prostatic sector number ‘x’, Bla = bladder, D_0.1cc_ and D_2cc_ = the doses received by the hottest 0.1 cc and 2 cc of the volume, respectively. Foci base = γH2AX/53BP1 foci count at baseline (pre-treatment (t = 0 h)). Foci R t = ratio of foci count at time point t relative to baseline (0 h). EPIC (x) = change in EPIC score from baseline; positive values reflecting improvement, negative values deterioration and 0 no change. * For space consideration all *ρ* values are presented as percentages

**Fig. 7:**
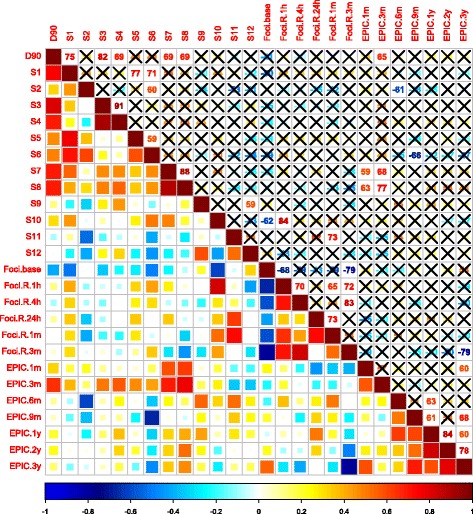
Sexual symptoms-related cross-correlation matrix of Spearman’s correlation coefficients (ρ). Upper triangle; *ρ* values with insignificant correlation (*p* ≤ 0.05) marked with an X. Lower triangle; colour coded coefficients. S (x) = prostatic sector number ‘x’. Foci base = γH2AX/53BP1 foci count at baseline (pre-treatment (t = 0 h)). Foci R t = ratio of foci count at time point t relative to baseline (0 h). EPIC (x) = change in EPIC score from baseline; positive values reflecting improvement, negative values deterioration and 0 no change. * For space consideration all *ρ* values are presented as percentages

Statistical correlation testing was conducted to explore potential signals of associations between the biological, patient − reported symptoms and dosimetric parameters using Spearman rank correlation coefficients. The results are presented in Figs. 5, 6 and 7. Background foci counts as well as the ratios at time point (t) relative to background (foci count prior to brachytherapy) are presented. Strong positive correlations were observed between mean foci counts at 24 h post-implantation and bowel toxicity at 1y and 2y, Fig. 5. This suggest that the larger the number of foci developed 24 h post-implant the more bowel symptoms the patients experienced. Although not statistically significant, a trend of positive correlations was also observed between the maximum dose to the rectum (rectum D_0.1cc_ and D_2cc_) and late bowel toxicity. Several sector doses also correlated with early γH2AX/53BP1 foci counts (see Figs. 5, 6 and 7) but these correlations were not significant.

## Discussion

By use of DNA DSB markers γH2AX and 53BP1, gamma-ray induced DSBs in peripheral blood lymphocytes of prostate cancer patients undergoing brachytherapy with ^125^I seeds can be readily visualised and quantified. X-ray induced DSBs have been detected numerous times in patients receiving EBRT [8, 9, 27, 28]. Moreover, in the field of nuclear medicine, Lassmann and colleagues have investigated the kinetics of γH2AX/53BP1 foci in peripheral leukocytes and lymphocytes in thyroid cancer patients being administered either ^177^Lu or ^131^I up to 144 hours post-infusion [12, 29, 30]. However, to our knowledge, this is the first study to demonstrate elevated DSBs in circulating lymphocytes in permanent prostate brachytherapy treatment and to study the kinetics of foci production in relation to late toxicity.

Lymphocytes within the peripheral blood circulation exist in equilibrium with large reservoirs of lymphocytes within the lungs and within extra vascular lymphatic tissue such as lymph nodes, the spleen and Peyer’s patches [31, 32]. Using ex-vivo FITC-labelled lymphocytes injected intravenously into sheep, Andrade et al. found these peripherally derived lymphocytes reached equilibrium with exchange to lymph tissue four hours post-injection [33]. Thus, at any given time, foci numbers measured in peripheral lymphocytes is a combination of both un-irradiated lymphocytes as well as those which have been irradiated by passing near the prostate at a range of earlier times. Redistribution of PBLs between intra and inter vascular spaces would be expected to reduce observed mean foci counts and increase the number of undamaged cells observed at early time points.

As expected, in the first hours post-implant an increase in foci count is observed, as levels of damage within the cells build up until the rate of repaired foci matches the induction of new damage by the radionuclide source. This process would be expected to reach equilibrium in a timeframe similar to that seen for DSB repair and PBL redistribution, both of which are typically on the order of a few hours. However, continual increases in DSB foci level are seen out to 24 hours and even four weeks post-implantation.

The continued increase to multiple weeks is particularly significant as by this time decay has reduced the activity of sources by more than a quarter. This persistent increase in damage is indicative of either extremely long-lived DNA damage within a proportion of PBL, or another effect increasing the rate of DSB induction even as the source decays. Foci kinetics at extremely long time points are poorly studied in lymphocytes, while the existence of fast and slow components of DNA repair are well established with reports of slow repair with half-lives of 38 hours [11]. It is possible to speculate that the current study is highlighting a proportion of DSBs repaired even slower or which totally failed to repair. Similarly, the effect of radiation on the distribution of lymphocytes between vascular and extra vascular − compartments is unknown. It is increasingly recognised that radiation derives an inflammatory response increasing lymphocytic infiltration in target tissues [34]. Any increase in PBL flow through the prostate would lead to a greater effective dose being seen by the lymphocytes, increasing the number of damaged cells and average foci numbers. Determining which of these effects dominates PBL foci kinetics requires further study.

One advantage DSB foci counting provides is that the dose received by the different structures (via DSBs) is over an integrated time post-implantation, whereas the post-treatment planning CT is a single snap shot of a patient’s physiology weeks later. The conventional method uses post-implant dose to predict radiation induced damage. An alternative method to assess radiation induced damage directly may lie in the ratio of foci observed between 0 h (and 4 h) and 24 h post − seed implantation.

In this study, positive correlations were found between increased early γH2AX/53BP1 foci production (24 h) and late bowel toxicity (EPIC 1y) (*p* = 0.035), EPIC 2 y (*p* = 0.001). Similar positive correlations were observed between increased foci count at 1 m relative to baseline and late bowel toxicity ((EPIC 1 y (*p =* 0.046), EPIC 2 y (*p =* 0.0189)).

Moreover, urinary toxicity positively correlated with the increase in doses received by prostatic base (sector 3, 4), D_90%_ and the maximum dose received by the hottest 2 cc of the bladder. These observations are in agreement with results published by Hathout et al. that the dose received by the bladder neck had a strong prognostic power for both early and late toxicity [35]. It may be that the region of bladder irradiation plays an important role in predicating toxicity alongside both total dose and volume irradiated. Urethral toxicity is likely to dominate urinary toxicity, especially in brachytherapy patients [36]. For bladder and rectal doses, it’s expected that these organs will move and change position quite frequently during treatment, for example, due to bladder filling and emptying [37] and small bowel peristalsis, potentially significantly altering the prescribed dose received to either or both organs. Prostatic sector 6 dose positively correlated with increase in sexual symptoms at all times. Significant strong positive correlations were found between late and early urinary symptoms (Fig. 6). Similar correlations were also found for bowel toxicity (Fig. 5) but not for sexual symptoms (Fig. 7).

Several articles have demonstrated the association between late normal tissue toxicity after EBRT and insufficient repair of DNA double − strand breaks in breast, head and neck and prostate cancer [38, 39]. In a study investigating DNA damage response as a possible risk factor for late toxicity in prostate cancer (*n* = 61), van Oorschot et al. found no significant differences between patient’s γH2Ax immediately after RT [40]. However, significant differences in foci decay (at 24 h post-irradiation) were observed between radio-sensitive and radio-resistant prostate cancer patients [40]. This implies that less efficient repair of radiation − induced DSBs may contribute to the development of late toxicity. These observations have great potential in the field of personalized medicine as for many years different tests have been proposed to investigate individual patient sensitivity to RT.

Several studies have shown that γH2AX could be used for in-vivo dose estimation for EBRT [27, 28]. In contrast with EBRT, the dose delivered in brachytherapy is more confined to the prostate and it accumulates over a period of time. Moreover, in general, smaller patient’s volumes are exposed to radiation compared to EBRT. Therefore, unsurprisingly our results in this study for brachytherapy indicate a lack of correlation between foci counts at different time points and dosimetric measures as the radiobiological effect is different compared to EBRT.

As early identification of patients with high risk of toxicity is of clinical importance, we investigated initial DNA damage foci production in relation to late toxicity. Strong positive correlations were found between early foci production ratios (relative to baseline) and late rectal toxicity. If this finding is validated in future clinical studies, this assay could have clinical utility as methods to reduce rectal dose using, for example, peri-rectal hydrogel spacers for patients with high levels of circulating DSBs at early time-points after seed implant [41].

The authors acknowledge the limitations of the study with the small number of patients and the multiple comparisons conducted. There are many methods to adjust for multiple comparisons (e.g. Bonferroni correction, Ducan’s multiple range tests), however, it is overly conservative to apply these methods in the current exploratory study [42]. The results from this study can be used to help to generate hypotheses for testing in larger clinical trials. Furthermore, future investigations on the relationship between DNA damage markers γH2AX/53BP1 and other markers e.g. citrulline, ceramide, cytokines (CXCL1, CXCL6, CXCL8, CXCL10, TNFα)), at specific time points may be necessary to gain more insight into the mechanisms responsible for the observed PBL foci kinetics and the associated toxicities.

## Conclusion

For the first time, we have demonstrated the detection and temporal changes of γH2AX/53BP1 DSB foci in peripheral blood lymphocytes for men treated with ^125^I permanent prostate brachytherapy. Radiation − induced γH2AX/53BP1 levels continue to increase for four weeks after seed implant and appear to reach equilibrium at less than 3 m where a clear repair signal was detected. In this small cohort of patients, levels of DSBs 24 h post-PPB seed implant positively correlated with late bowel toxicity. However, these findings require validation in larger patient cohorts to detect correlations with greater certainty.

## Additional files

Additional file 1: Figure S1. Twelve-sector analysis created by dividing the prostate into three equal lengths along the cranio-caudal axis to form the base, mid-gland, and apex and then subdivide with vertical and horizontal plans generating right/left posterior sectors (reused from (21) with permission from AB Mohamed Yoosuf). (PDF 61 kb)
Additional file 2: Figure S2. Top; dose rate (cGy/h) as a function of time since implant for ^125^I monotherapy plotted using the equation, $$ Dose\  rate\ (t)= m P D\left(\frac{ \ln 2}{t_{\raisebox{1ex}{$1$}\!\left/ \!\raisebox{-1ex}{$2$}\right.}}\right){.2}^{\left(\frac{t}{t_{\raisebox{1ex}{$1$}\!\left/ \!\raisebox{-1ex}{$2$}\right.}}\right)} $$, where *t* is the elapsed time, *mPD* is the minimum peripheral dose (=145 Gy for ^125^I) and $$ {t}_{\raisebox{1ex}{$1$}\!\left/ \!\raisebox{-1ex}{$2$}\right.} $$ is the half-life (=59.43 days for ^125^I). Bottom; the time required to deliver relative fraction of the prescribed dose, $$ Fractional\  dose(t)=1-{e}^{-\left(\frac{t. \ln 2}{t_{\raisebox{1ex}{$1$}\!\left/ \!\raisebox{-1ex}{$2$}\right.}}\right)} $$. Reference: Dale RG. The applications of the linear-quadratic dose effect equation to fractionated and protracted therapy. Br J Radiol 1985; 58: 515–28. (PDF 178 kb)

## Abbreviations

ADT: Androgen deprivation therapy
Cc: Cubic centimetres
DSB: Double strand break
EPIC: Expanded Prostate Cancer Index Composite
PPB: Permanent prostate brachytherapy
PSA: Prostate-specific antigen
Qol: Quality of life
