# Supplementary figures and images for: Prostate cancer treated with brachytherapy; an exploratory study of dose-dependent biomarkers and quality of life
Source: Radiat Oncol. 2017 Mar 14;12:53. doi: 10.1186/s13014-017-0792-1 (PMC5348795; doi:10.1186/s13014-017-0792-1)

Additional figure 1

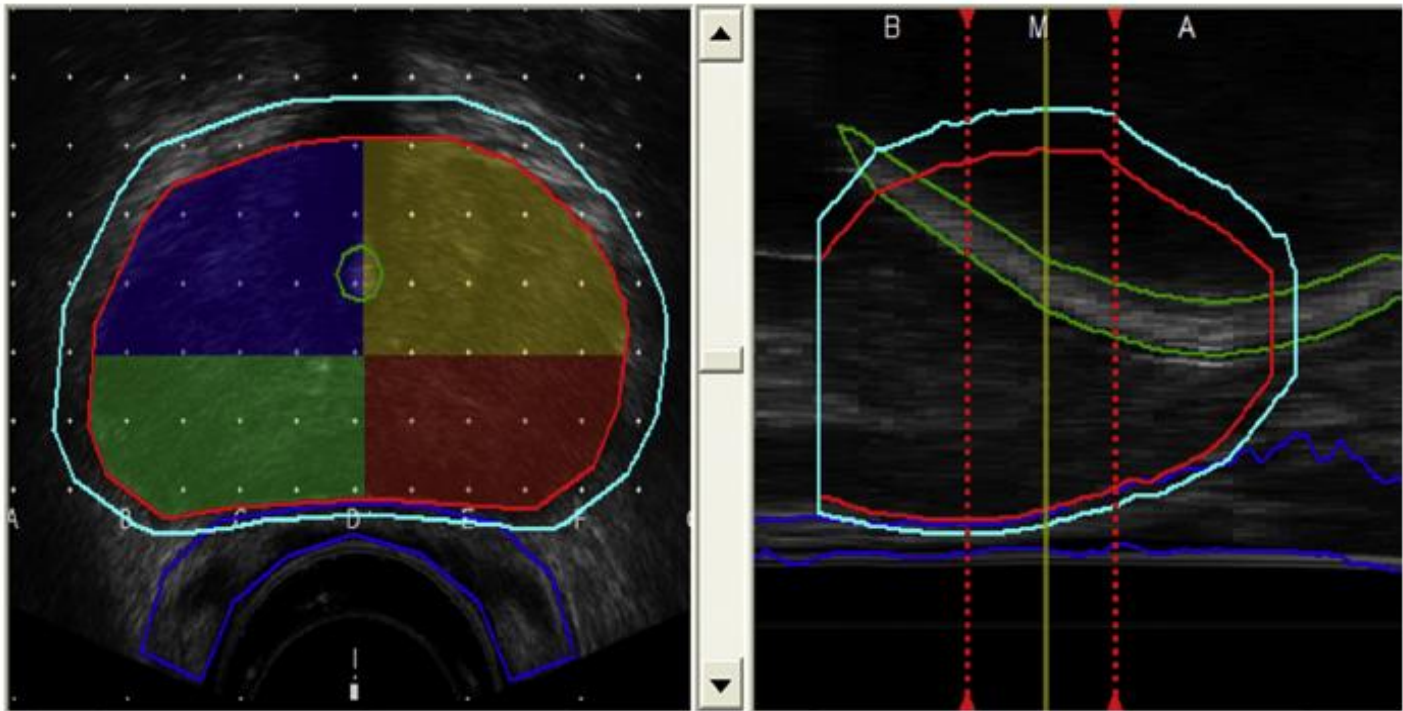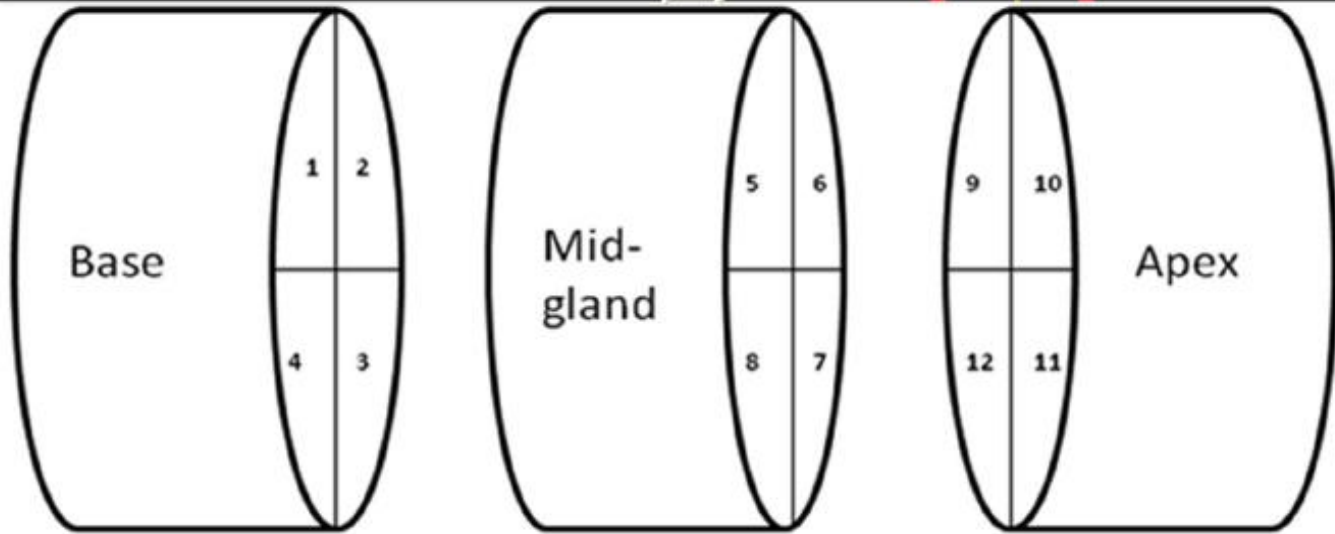

Supplement: Additional file 1: Figure S1. — Twelve-sector analysis created by dividing the prostate into three equal lengths along the cranio-caudal axis to form the base, mid-gland, and apex and then subdivide with vertical and horizontal plans generating right/left posterior sectors (reused from (21) with permission from AB Mohamed Yoosuf). (PDF 61 kb) [file 13014_2017_792_MOESM1_ESM.pdf]

Additional Figure 2

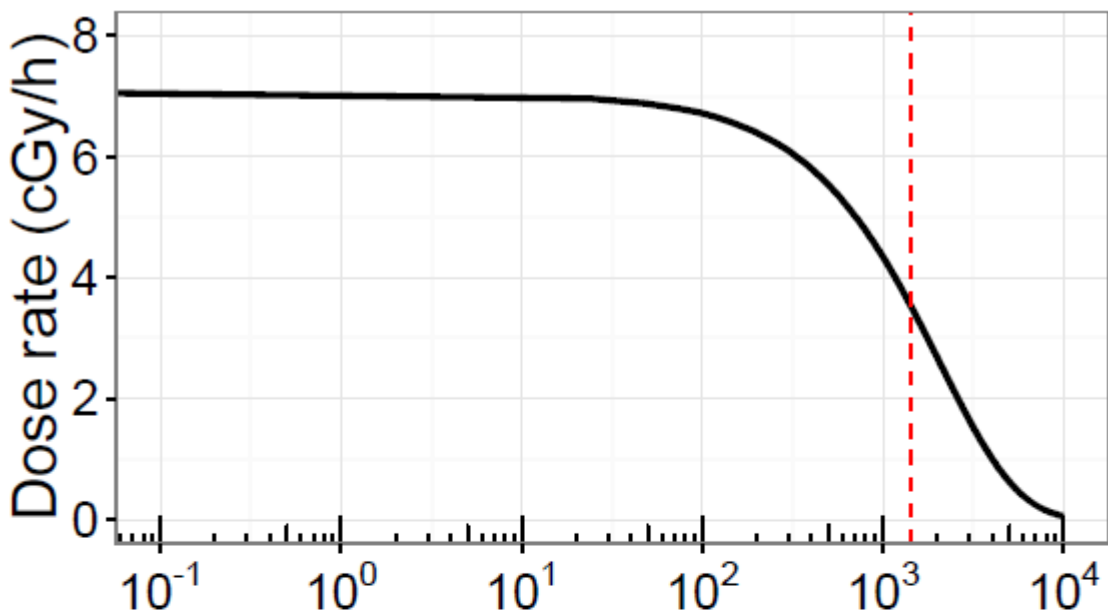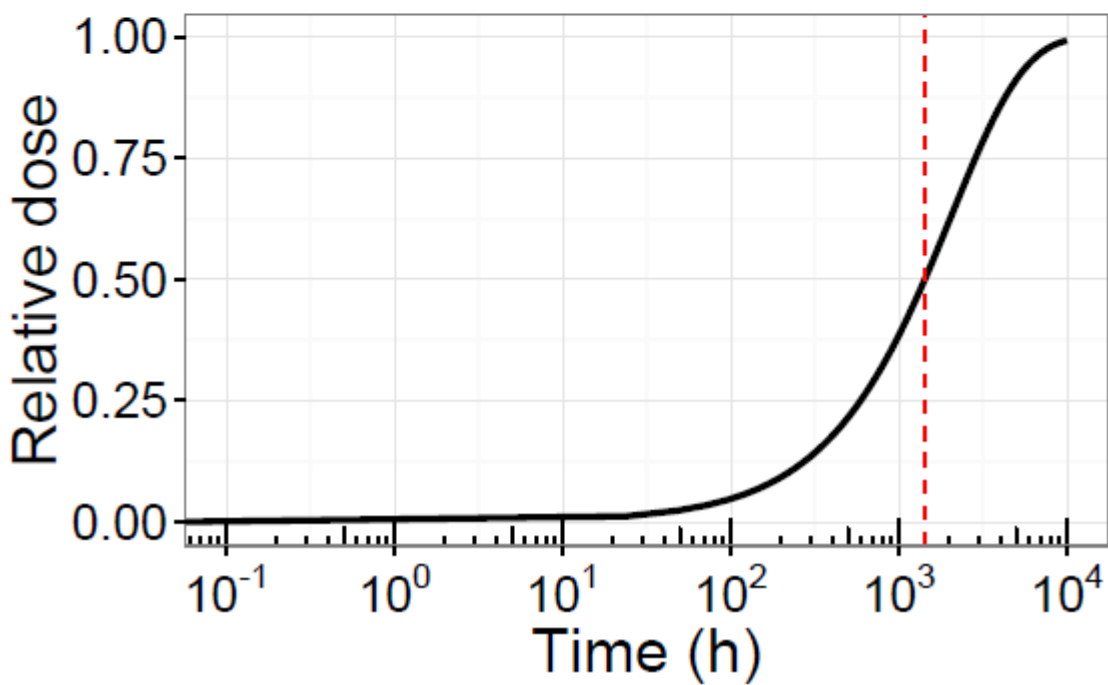

Supplement: Additional file 2: Figure S2. — Top; dose rate (cGy/h) as a function of time since implant for 125I monotherapy plotted using the equation, $$ Dose\ rate\ (t)= m P D\left(\frac{ \ln 2}{t_{\raisebox{1ex}{$1$}\!\left/ \!\raisebox{-1ex}{$2$}\right.}}\right){.2}^{\left(\frac{t}{t_{\raisebox{1ex}{$1$}\!\left/ \!\raisebox{-1ex}{$2$}\right.}}\right)} $$Doseratet=mPDln2t12.2tt12, where t is the elapsed time, mPD is the minimum peripheral dose (=145 Gy for 125I) and $$ {t}_{\raisebox{1ex}{$1$}\!\left/ \!\raisebox{-1ex}{$2$}\right.} $$t12 is the half-life (=59.43 days for 125I). Bottom; the time required to deliver relative fraction of the prescribed dose, $$ Fractional\ dose(t)=1-{e}^{-\left(\frac{t. \ln 2}{t_{\raisebox{1ex}{$1$}\!\left/ \!\raisebox{-1ex}{$2$}\right.}}\right)} $$Fractionaldoset=1−e−t.ln2t12. Reference: Dale RG. The applications of the linear-quadratic dose effect equation to fractionated and protracted therapy. Br J Radiol 1985; 58: 515–28. (PDF 178 kb) [file 13014_2017_792_MOESM2_ESM.pdf]
